# Autonomic arousal to emojis: Electrodermal activity and respiratory sinus arrhythmia analysis

**DOI:** 10.14814/phy2.70225

**Published:** 2025-04-11

**Authors:** Deeksha Patel, Abhinav Dixit, Om Lata Bhagat

**Affiliations:** ^1^ Cognitive Neurophysiology Laboratory, Department of Physiology All India Institute of Medical Sciences Jodhpur Rajasthan India; ^2^ Autonomic Function Test Laboratory, Department of Physiology All India Institute of Medical Sciences Jodhpur Rajasthan India

**Keywords:** autonomic nervous system, electrodermal Activity, emojis, emotional Stroop task, respiratory sinus arrhythmia

## Abstract

Emojis have become vital in virtual communication, mimicking facial expressions, and gestures to convey emotions. This study investigates their influence on emotional perception and autonomic responses during non‐face‐to‐face interactions. Hundred healthy participants (50 men, 50 women) aged 18–40 years were recruited. The emotional Stroop task (EST), incorporating emojis with congruent and incongruent emotional words, was used to assess emotional valence. Electrodermal activity (SCL and SCR amplitude) and respiratory sinus arrhythmia (RSA) were measured during task performance. Both SCL and SCR amplitudes were significantly higher in congruent and incongruent blocks compared to the neutral block, indicating increased sympathetic activity. RSA was significantly lower in these blocks, reflecting parasympathetic withdrawal. These findings suggest heightened autonomic responses during emoji‐word EST. Emojis effectively evoke autonomic responses, influencing both sympathetic (EDA) and parasympathetic (RSA) systems. No gender differences were observed in autonomic reactions to emojis. This study highlights the potential of emojis as stimuli for emotion, cognitive and physiological research.

## 1 INTRODUCTION

In digital communication, the absence of facial expressions and gestures can hinder the emotional clarity of messages. Emojis were introduced to address this gap, serving as visual tools to convey emotional context (Bakker et al., 2020). Their ability to evoke neural responses similar to those elicited during face‐to‐face interactions makes them effective in enhancing message comprehension (Ruiz‐Robledillo et al., 2019a, 2019b; Thompson et al., 2018a, 2018b).

Neuroimaging studies, using fMRI have identified key brain regions involved in processing emotional stimuli. Whereas, EEG studies examining event related potentials showed that components like the late positive potential (LPP) are sensitive to the valence (positive or negative) and arousal (intensity) of emotional stimuli (Kreibig, 2010; Pizzagalli et al., 2020). These findings highlight the temporal and spatial dynamics of emotion processing in the brain.

Cognitive science research complements neuroimaging studies by exploring the theoretical frameworks underlying emotional processing. One prominent model is the valence‐arousal model, which posits that emotional stimuli are evaluated along two primary dimensions: valence (positive or negative emotional value) and arousal (intensity of activation). High‐arousal emotions, such as fear or anger, are processed more rapidly and robustly, engaging attentional and memory systems due to their evolutionary significance. Conversely, low‐arousal emotions, such as sadness, elicit subtler cognitive, and physiological responses, reflecting the dynamic interplay between the salience and relevance of emotional stimuli (Lang et al., 2018).

Beyond neural mechanisms, physiological markers such as electrodermal activity (EDA) and respiratory sinus arrhythmia (RSA) provide valuable insights into the autonomic correlates of emotional processing. EDA reflects sympathetic nervous system activity and comprises tonic (skin conductance level, SCL) and phasic (skin conductance responses, SCR) components, both of which are influenced by the intensity and type of emotional stimuli (Ziegler et al., 2020). RSA, regulated by the parasympathetic nervous system, measures heart rate variability associated with breathing and offers an index of vagal control during emotional arousal (Feurer et al., 2020a, 2020b; Thayer & Lane, 2009). These markers are crucial for understanding how the autonomic nervous system responds to emotional stimuli and how congruence between stimuli and affective states modulates these responses.

Gender differences in emotional processing and autonomic responses have also been documented. Men tend to exhibit higher electrodermal activity due to differences in sweat gland morphology and activity. In contrast, women often display greater sensitivity to emotional stimuli, possibly reflecting heightened engagement with social and affective cues (Martinez‐Selva et al., 2018). Neuroimaging studies suggest that women may show stronger amygdala activation in response to emotionally salient stimuli, while men often rely more on cortical regions for emotion regulation (Guyer et al., 2018). These differences underscore the importance of considering gender as a factor in studies of emotional processing and autonomic reactivity (Kreibig, 2010; Ziegler et al., 2020).

Despite the growing use of emojis in digital communication, their impact on emotional processing, particularly in terms of psychophysiological responses, remains underexplored. While the emotional Stroop task (EST) has effectively elicited emotional arousal in prior research, the unique role of emojis in modulating both sympathetic and parasympathetic nervous system activity has yet to be fully understood. Additionally, gender differences in these responses have not been sufficiently investigated. This study aims to fill these gaps by examining how emojis influence emotional processing, as measured by changes in sympathetic and parasympathetic nervous system activity, and by exploring the role of gender differences in these emotional responses.

## 2 MATERIALS AND METHODS

The study was conducted in the Cognitive Neurophysiology Laboratory and initiated after approval from the institutional ethics committee of All India Institute of Medical Sciences Jodhpur (reference no. AIIMS/IEC/2018/796). A total of 100 healthy participants (50 males and 50 females) were recruited within the age group of 18–40 years (mean ± SD—27.87 ± 5.37 years), and participants gave written informed consent for the experiment and to publish images taken while performing the experiment. Selection criteria encompassed educational attainment up to the fifth grade, proficiency in computer utilization, and a documented absence of medical conditions impacting the autonomic nervous system (ANS) and respiratory pathophysiology. The Psychological General Well‐Being Index (PGWBI) was used to assess the emotional stability of individuals. Participants with a raw index score (RIS) of at least 73 were considered emotionally, physically, and mentally healthy and were included in the study.

### 2.1 Procedure

Participants were invited to the laboratory during the mid‐morning period (between 9:00 a.m. and 1:00 p.m.) to minimize the influence of circadian rhythms. Environmental conditions within the laboratory were carefully controlled, maintaining a thermoneutral temperature and ensuring a noise‐free ambiance to mitigate potential sources of apprehension. Upon arrival, participants were provided with verbal instructions regarding the study procedures, followed by a preliminary training session. Electrodermal activity (EDA) and respiratory sinus arrhythmia (RSA) parameters were recorded utilizing the Biopac MP150TM system (BiopacSystem, Inc. (C) Copyright 2000‐2014, USA). For EDA measurement, disposable silver/silver chloride disk electrodes (EL507, 3 M, India Ltd.) paired with EL101 isotonic gel were positioned on the thenar and hypothenar regions of the nondominant hand. The BioNomadixTM wireless EDA recording module was affixed to the wrist and linked to the electrodes, while a PPGED‐RTM skin conductivity module amplified the electrical signals. Simultaneously, electrocardiogram (ECG) signals and respiratory data from lead II were recorded using the RMSTM physiograph (Recorders and Medicare system (P) Ltd./POLYG1311020), with a respiratory belt (RMSTM) placed across the participant's chest at the fifth intercostal space. These systems were integrated via a UIM100CTM signal preamplifier with connectivity to a computer equipped with the Biopac MP150TM system. Real‐time visualization of the data was facilitated using AcqknowledgeTM software version 4.4. Baseline recordings of ECG, respiratory rate, and skin conductance were obtained over 5 min and were maintained throughout the experimental task. Data collection was synchronized with task presentation, and EDA, ECG, and respiratory signals were sampled at a rate of 2 kHz. RSA analysis necessitated ECG lead II and respiration signals, with RSA values computed as the difference between maximum and minimum RR intervals per breath. To streamline computational processing, EDA data were resampled and analyzed at 200 samples per second. The phasic component of EDA was extracted from skin conductance level (SCL) data.

**FIGURE 1:**
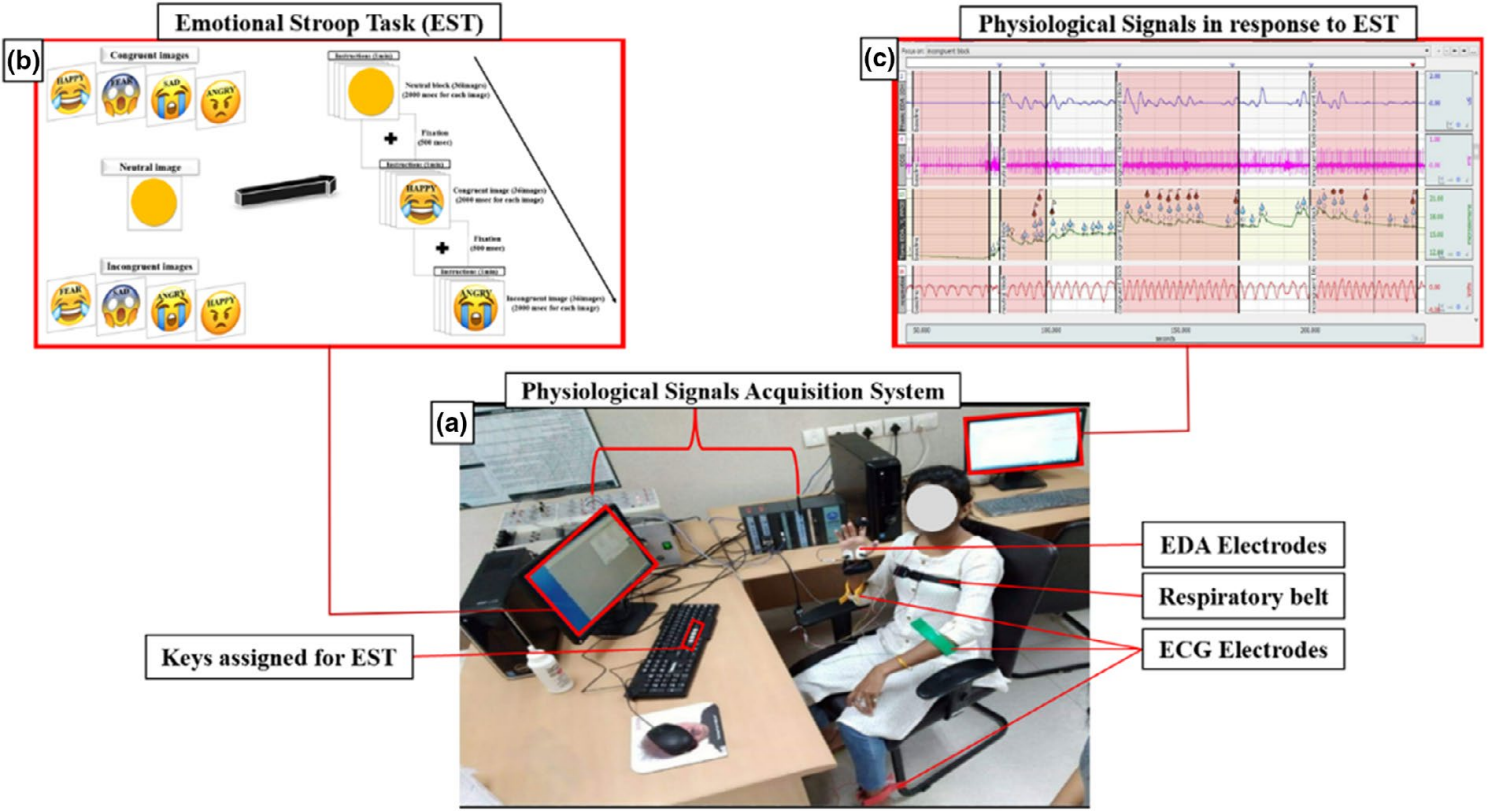
Study protocol. (a) physiological signal acquisition system. (b) emotional Stroop task. (c) physiological signals in response to EST A. *Participant has given written consent to publish the picture.

The emotional Stroop task consisted of 108 trials divided into neutral, congruent, and incongruent blocks, each with 36 trials (Figure 1). Neutral blocks featured emojis without facial expressions (yellow circles), while congruent blocks paired emojis with facial expressions matching the emotional word, such as a happy emoji with “happy.” In incongruent blocks, mismatched combinations like an angry emoji with “happy” were presented. Each block included nine trials for each of the four target emotions—happiness, sadness, anger, and fear—to ensure balance. Participants identified the facial expression of the emoji while ignoring the emotional word, responding quickly, and accurately using designated keys. Stimuli were displayed via SuperLab 5.0, with words in black, bold Times New Roman font above the emoji's eyes, ensuring clear visibility. Emojis were selected based on normative datasets, such as Rodrigues et al. (2018), ensuring alignment with target emotions through controls on valence, arousal, and familiarity. Usage trends from Unicode reports ensured recognizability, making emojis intuitive stimuli for capturing emotional processing. Words, selected from databases like ANEW, were matched to emoji emotions using strict controls on variables like valence and familiarity, validated through independent participant ratings for consistency. This design ensured fluency and precision in examining emotional interference.

### 2.2 Statistical analysis

The data obtained were analyzed using SPSS version 20 software (IBM). Data were found to be normally distributed in normality tests and therefore expressed as mean ± SD. Statistical analysis was performed using repeated measures ANOVA followed by a post hoc Tukey test for comparison between all three blocks. Sex differences in autonomic responses were assessed using an independent sample *t*‐test. A *p* value ≤0.05 was considered significant.

## 3 RESULTS

### 3.1 Electrodermal activity (sympathetic nervous system activity)

**FIGURE 2:**
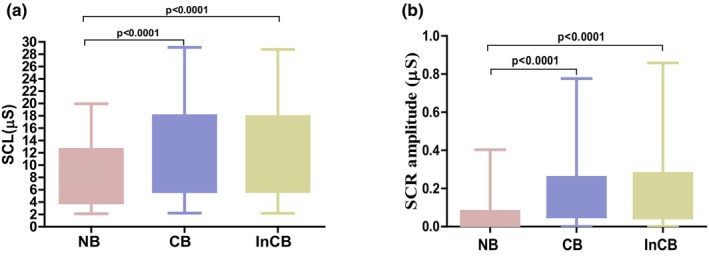
Box and whisker plot showing (a) skin conductance level (SCL, μs) and (b) skin conductance response amplitude (SCR amp., μs) across neutral (NB), congruent (CB), and incongruent (InCB) blocks during the emotional Stroop task. The box represents the interquartile range (IQR), and the whiskers extend to the minimum and maximum values within 1.5 times the IQR.

Electrodermal activity was calculated for neutral, congruent, and incongruent blocks. The mean skin conductance (SCL) was higher in the incongruent block (14.64 ± 6.73 μs) and in the congruent block (12.99 ± 6.26 μs) than in the neutral block (7.75 ± 4.93 μs) (Figure 2a). The SCL was higher in all three blocks, in men (18.65 ± 6.20 μs, 16.52 ± 6.07 μs, and 10.16 ± 4.99 μs, respectively) compared to women (10.63 ± 4.50 μs, 9.46 ± 4.13 μs and 5.33 ± 3.52 μs). Differences in SCL were statistically significant for neutral, congruent, and incongruent blocks (*p* < 0.001).

An increase in skin conductance of more than 0.02 μs was considered a specific response. These specific responses were measured as skin conductance response amplitude (SCR amplitude). The average SCR amplitude was higher for the incongruent block (0.182 ± 0.168 μs) and the congruent block (0.158 ± 0.134 μs) than for the neutral block (0.021 ± 0.015 μs) (Figure 2b). SCR amplitude in men (0.238 ± 0.204 μs, 0.193 ± 0.156 μs, and 0.023 ± 0.014 μs) was higher than in women (0.126 ± 0.096 μs, 0.122 ± 0.096 μs, and 0.019 ± 0.017 μs) for all three blocks. Data were statistically significant for all three blocks (*p* < 0.001).

**FIGURE 3:**
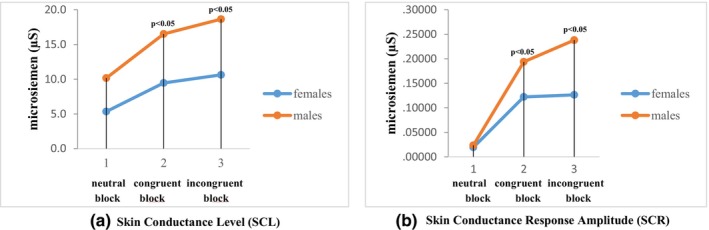
The average value of electrodermal activity parameters (a. SCL and b. SCR) among males and females while performing the EST. The vertical dashed line indicates the beginning of a neutral, congruent, and incongruent block of EST.

In addition, the absolute and percentage changes were calculated and compared between the two sexes (Figure 3). The result of the independent sample *t*‐test showed that the difference between the two genders in the absolute change in electrodermal activity parameters during EST was statistically significant (the SCL‐congruent condition [*t* (98) = 3.755, *p* < 0.05], the incongruent condition [*t* (98) = 5.139, *p* < 0.05] and for the SCR congruent condition [*t* (98) = 2.619, *p* < 0.05], incongruent condition [*t* (98) = 3.368, *p* < 0.05]). In contrast, the percentage changes in electrodermal activity parameters (SCL and SCR amp.) were similar in men and women during congruent and incongruent blocks. This suggests that although sympathetic responses to emojis are different in both genders, the amplitude of the response is similar between them. This reaffirms that emojis have the ability to evoke similar sympathetic reactions in men and women.

### 3.2 Respiratory sinus arrhythmia (RSA)—Parasympathetic nervous system activity

**FIGURE 4:**
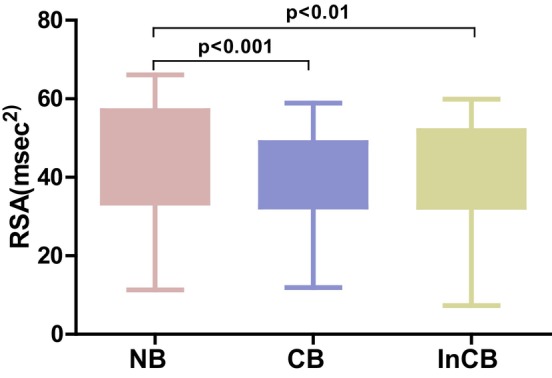
Box and whisker plot showing respiratory sinus arrhythmia (RSA, ms^2^) across neutral (NB), congruent (CB), and incongruent (InCB) blocks during the emotional Stroop task. The box indicates the interquartile range (IQR), and the whiskers extend to the minimum and maximum values within 1.5 times the IQR.

To calculate RSA, the difference between the maximum and minimum RR intervals per breath was calculated for neutral block, congruent block, and incongruent block (Figure 4). The mean RSA was found to decrease during incongruent block (36.47 ± 10.53 msec^2^) and congruent block (39.40 ± 10.15 msec^2^) compared to neutral block (48.66 ± 10.27 msec^2^). The average RSA value was lower in men (35.72 ± 12.17 msec^2^, 37.95 ± 12.18 msec^2^, and 49.72 ± 12.90 msec^2^) compared to women (37.74 ± 9.88 msec^2^, 41.37 ± 10.17 msec^2^, and 50.82 ± 10.12 msec^2^). The difference in RSA was statistically significant (*p* < 0.001).

**FIGURE 5:**
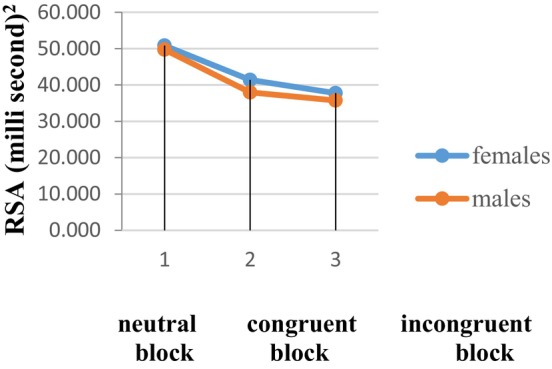
The average value of respiratory sinus arrhythmia (msec^2^) among males and females while performing the EST. The vertical dashed line indicates the beginning of the neutral, congruent, and incongruent block of EST.

Further analyzing gender, the result of the independent sample *t*‐test showed that the difference in absolute change and percentage change in RSA score between men and women were not statistically significant (*p* > 0.05). Although the combined data from men and women showed that emojis can elicit parasympathetic responses, we found no difference when comparing between sexes (Figure 5).

## 4 DISCUSSION

This study investigated how emojis, as digital communication tools, influence autonomic nervous system (ANS) responses, particularly through sympathetic and parasympathetic pathways. We assessed electrodermal activity (SCL and SCR) and respiratory sinus arrhythmia (RSA) during an emoji‐word emotional Stroop task (EST) in 100 healthy participants (50 men and 50 women). The findings indicated a pronounced modulation of autonomic function during exposure to emotional emoji‐word stimuli, with sympathetic activation and parasympathetic withdrawal being evident, particularly in the incongruent blocks of the task.

Electrodermal responses, including skin conductance level (SCL) and skin conductance response (SCR), served as markers for sympathetic nervous system (SNS) activation. The results indicated significantly heightened sympathetic arousal in both congruent and incongruent blocks of the EST, where emotional stimuli (emoji‐word combinations) were presented. Specifically, SCL and SCR amplitudes were significantly higher in the incongruent and congruent blocks compared to the neutral block. This finding suggests emojis can act as potent emotional stimuli, evoking heightened autonomic responses. Our results align with previous studies examining emotional and autonomic responses to various stimuli. Thompson et al. (2018a, 2018b) demonstrated increased electrodermal activity when participants were exposed to emoticons used in messages, indicating that emoticons—similar to emojis—effectively trigger autonomic responses (Thompson et al., 2018a, 2018b). Additionally, the increased sympathetic response during incongruent blocks can be attributed to cognitive interference, as participants were tasked with identifying emoji emotions while ignoring conflicting word stimuli. This cognitive load, coupled with emotional incongruence, is likely to intensify emotional arousal and attention, leading to an elevated sympathetic response. Furthermore, the heightened sympathetic activation observed in the congruent block, while less pronounced than in the incongruent condition, also supports the hypothesis that emojis, regardless of congruency, elicit emotional responses. Emojis, even when congruent with the emotional word, appear to augment autonomic arousal, potentially clarifying the emotional intention behind the communication. Consistent with our findings, Ruiz‐Robledillo et al. (2019a, 2019b) highlighted that emoticons can heighten arousal by modulating attention and emotional processing (Ruiz‐Robledillo et al., 2019a, 2019b). Furthermore, some studies have suggested that the emotional context provided by emojis in digital communication mimics real‐life interactions, where facial expressions would otherwise play a crucial role in emotional expression (D'Acquisto et al., 2021). A study by D'Acquisto et al. (2021) also suggested that the addition of emojis enhances communication by signaling emotional tone in the absence of facial expressions.

In contrast to sympathetic activation, parasympathetic nervous system (PNS) responses were assessed using respiratory sinus arrhythmia (RSA). Our findings revealed a significant reduction in RSA during both congruent and incongruent blocks compared to the neutral block, indicating parasympathetic withdrawal during exposure to emotional emojis. This reduction in RSA, particularly in the incongruent blocks, reflects heightened emotional arousal and the body's natural tendency to shift toward a fight‐or‐flight state under stressful conditions (Feurer et al., 2020a, 2020b). The observed RSA decline during incongruent blocks is consistent with the notion that emotional stress leads to the withdrawal of parasympathetic influence, thereby accelerating heart rate and respiratory rate to prepare for a heightened state of alertness. The pattern of RSA reduction in response to emotional stimuli has been documented in other emotional Stroop task studies, where emotional incongruence and conflict lead to a decreased parasympathetic response. Mathewson et al. (2017) found that RSA decreased during emotionally challenging tasks, particularly when the emotional stimuli induced cognitive conflict (Mathewson et al., 2017). This supports the current study's findings that emojis—especially when paired with incongruent words—engage the sympathetic nervous system and decrease parasympathetic modulation.

Our analysis revealed notable gender differences in sympathetic responses, with men showing significantly higher SCL and SCR amplitudes than women across all blocks. These findings are in line with previous studies indicating that men tend to exhibit greater sympathetic activation due to differences in sweat gland activity and overall skin conductance (Martinez‐Selva et al., 2018). Martinez‐Selva et al. (2018) explored gender differences in electrodermal activity, noting that men typically demonstrate stronger electrodermal responses in comparison to women, potentially due to morphological differences in sweat gland density. However, it is worth noting that despite these gender differences in autonomic activity, the overall pattern of response to emojis was similar across sexes. This suggests that both men and women exhibit similar autonomic reactivity to emotional emojis, reinforcing the idea that emojis serve as effective emotional stimuli across genders. Interestingly, no significant gender differences were observed in RSA. Although men showed lower RSA than women during the neutral block, the reduction in RSA during emotional induction (whether congruent or incongruent) was comparable across genders. This suggests that while gender differences in sympathetic responses may exist, parasympathetic modulation in response to emotional stimuli does not significantly differ between men and women. This finding aligns with studies by Feurer et al. (2020a, 2020b), which indicated that although girls tend to show stronger RSA suppression in high‐stress environments, the overall trend of parasympathetic withdrawal in response to emotional stimuli is similar across genders in other contexts (Fekri et al., 2022; Feurer et al., 2020a, 2020b).

The results of this study build upon existing research into emotional processing and autonomic responses. Emojis have been shown to influence emotional perception and physiological responses in various contexts, such as social interactions and marketing communications (Zong et al., 2022). Our study is among the first to examine the specific effects of emojis on autonomic nervous system activity during a controlled emotional Stroop task. The observed autonomic responses—heightened sympathetic activity and diminished parasympathetic modulation—reflect the potent emotional impact of emojis and their ability to evoke strong physiological reactions, similar to those elicited by facial expressions in face‐to‐face communication (Thompson et al., 2018a, 2018b). Further, the study's results resonate with the findings of Thompson et al. (2018a, 2018b), which suggested that emoticons increase electrodermal activity and modulate the emotional impact of messages. Our study extends this work by exploring the specific contributions of congruent and incongruent emoji‐word combinations to sympathetic and parasympathetic activation. The incongruence between emoji and words in our task generated the strongest autonomic response, highlighting the emotional potency of such mismatches and their ability to increase cognitive load, stress, and emotional arousal (Kocielnik et al., 2020).

### 4.1 Limitations and future directions

While our study provides valuable insights into the autonomic responses to emojis, it is not without limitations. One limitation is the relatively small sample size (100 participants), which may constrain the generalizability of the findings to broader populations. Additionally, the emotional valence of emojis was not exhaustively explored, and future research should consider examining a wider range of emotional stimuli to better understand how different types of emojis (e.g., happy, sad, and angry) elicit distinct physiological responses. Further, this study focused on healthy adults, and exploring the effects of emojis on individuals with emotional or autonomic disorders (e.g., anxiety, myocardial infarction, or autonomic neuropathy) could yield important insights into the therapeutic implications of emojis in communication. Future studies should also incorporate longitudinal designs to investigate how prolonged exposure to emotional emojis impacts autonomic function over time, particularly regarding mental and physical health outcomes.

## 5 CONCLUSION

This study provides compelling evidence that emojis, as a form of digital communication, elicit significant autonomic nervous system responses, with increased sympathetic activation and decreased parasympathetic modulation. The findings suggest that emojis effectively modulate emotional perception, heighten arousal, and influence both emotional and physiological processes during non‐face‐to‐face interactions. These results have implications for understanding how digital communication tools, such as emojis, may affect emotional processing and health, particularly for vulnerable populations.

## AUTHOR CONTRIBUTIONS

Deeksha Patel: experimental design of the study, conceptualization, methodology, investigation, performed experiments/data collection, data analysis and interpretation, writing an original draft, reviewing, and editing. Abhinav Dixit: conceived the idea of the experiment, validation of data, supervision, and provided revision to the manuscript. Om Lata Bhagat: conceived the idea of the experiment, experimental design, validation of data, supervision, provided resources and access to the crucial research component, and Provided revision to the scientific content of the manuscript.

## FUNDING INFORMATION

No funding information provided.

## CONFLICT OF INTEREST STATEMENT

The authors declare that no conflict of interest, financial or otherwise, exists.

## ETHICS STATEMENT

All procedures performed in studies involving human participants were in accordance with the Code of Ethics of the World Medical Association (Declaration of Helsinki) and ethical standards of the institutional ethics committee of All India Institute of Medical Sciences (AIIMS), Jodhpur (reference no. AIIMS/IEC/2018/796).

## Data Availability

The data generated or analyzed during this study can be found within the published article and additional data will be available from the corresponding author on reasonable request.
